# Histone deacetylase inhibitors with high *in vitro* activities against *Plasmodium falciparum* isolates collected from Gabonese children and adults

**DOI:** 10.1038/s41598-019-53912-w

**Published:** 2019-11-22

**Authors:** Erik Koehne, Andrea Kreidenweiss, Rella Zoleko Manego, Matthew McCall, Ghyslain Mombo-Ngoma, Marcel Karl Walter Mackwitz, Finn K. Hansen, Jana Held

**Affiliations:** 1 Institute of Tropical Medicine, University Hospital Tübingen, Wilhelmstrasse 27, D-72074 Tübingen, Germany; 2 Centre de Recherches Médicales de Lambaréné, B.P. 242 Lambaréné, Gabon; 3 Department of Tropical Medicine, Bernhard Nocht Institute for Tropical Medicine & I. Dep of Medicine, University Medical Centre- Hamburg-Eppendorf, Bernhard-Nocht-Strasse 74, D-20359 Hamburg, Germany; 4 Institute of Pharmacy, Medical Faculty, University of Leipzig University, Brüderstrasse 34, D-04103 Leipzig, Germany

**Keywords:** Diseases, Drug development

## Abstract

Histone deacetylase (HDAC) enzymes are targets for the development of antimalarial drugs with a different mode of action to established antimalarials. Broad-spectrum HDAC-inhibitors show high potency against *Plasmodium falciparum*, but displayed some toxicity towards human cells. Inhibitors of human HDAC6 are new drug candidates with supposed reduced toxicity to human cells and favorable activities against laboratory *P. falciparum* strains. We investigated the potency of 12 peptoid-based HDAC-inhibitors against asexual stages of *P. falciparum* clinical isolates. Parasites representing different genetic backgrounds were isolated from adults and children with uncomplicated malaria in Gabon. Clinical studies on (non-HDAC-inhibitors) antimalarials, moreover, found lower drug efficacy in children, mainly attributed to acquired immunity with age in endemic areas. Therefore, we compared the *in vitro* sensitivity profiles of adult- and child-derived isolates to antimalarials (HDAC and standard drugs). All HDAC-inhibitors showed 50% inhibitory concentrations at nanomolar ranges with higher activities than the FDA approved reference HDAC-inhibitor SAHA. We propose peptoid-based HDAC6-inhibitors to be lead structures for further development as antimalarial chemotherapeutics. Our results further suggest no differences in activity of the tested antimalarials between *P. falciparum* parasites isolated from children and adults.

## Introduction

Malaria is caused by protozoan parasites of the genus *Plasmodium* and is the most important parasitic disease worldwide. *Plasmodium falciparum* - the most virulent species - has become resistant to nearly all of the antimalarial compounds that are in clinical use^1–4^. In 2008, first evidence of artemisinin-resistant parasites was reported in western Cambodia^1,2^. There is a growing fear that resistance to artemisinin will continue to spread, especially to Sub-Saharan Africa. To keep up with resistance development of *P. falciparum*, new treatment options are constantly needed and chemical scaffolds with a new mode of action are of particular interest as they are less prone to be affected by cross-resistances. Histone deacetylase inhibitors (HDACi) are new antimalarial lead compounds known to inhibit multiple life cycle stages of *P. falciparum*^5,6^.

In eukaryotes, deoxyribonucleic acid (DNA) is wrapped into tightly packed chromatin with the nucleosomes acting as the fundamental unit. Each nucleosome is composed of an octamer of two copies of four core histones. HDACs play an important role in the wrapping and unwrapping of DNA by increasing the affinity of histone octamers for DNA by removing acetyl groups from the side chain of specific lysine residues^7^. Inhibition of HDACs interferes with modulation of transcription, replication and DNA repair, and also the function of non-histone proteins^8^. Research on HDACi to target human diseases such as cancer has been ongoing for several decades and first compounds have entered the clinic as cancer therapies, proving the safety of this compound class for human use^7,9–12^. Recent data suggest HDACi as attractive drug candidates against parasitic diseases caused by *Trypanosoma, Toxoplasma, Schistosoma, Leishmania, and Plasmodium*^13–17^. The natural product apicidin was the first HDACi to be tested against *P. falciparum* and exhibited broad-spectrum antiprotozoal activity *in vitro* and *in vivo* in mice^18^. SAHA (suberoylanilide hydroxamic acid, vorinostat), romidepsin, belinostat, and panobinostat are all clinically approved HDACi used for cancer treatment and affect growth of various *Plasmodium* species including drug resistant *P. falciparum* strains^15^. Notably, HDACi were shown to be active against multiple life-cycle stages of *P. falciparum* including liver stages and gametocytes^12,19–21^. HDACi are promising lead structures for antimalarial drug development, but their use might otherwise be limited due to concomitant toxicity to human cells. This problem could be mitigated by developing inhibitors with relative or complete specificity towards plasmodial HDACs. In *P. falciparum*, up to six HDACs have been identified that share only a certain degree of sequence identity with human HDACs^22^. Little knowledge about structure and function of these enzymes in *P. falciparum* limits structure-based design of new inhibitors^23^. An alternative approach is to expand on human HDACi molecules, which are known to be less harmful to mammalian cells and drive their development towards parasite selectivity as well as anti-plasmodial activity. Selective inhibitors of human HDAC6 (hHDAC6), a class II enzyme, exert lower levels of cytotoxicity to human cells compared to HDAC class I inhibitors^24^. hHDAC6 targets in particular non-histone proteins (alpha-tubulin, Hsp90) and class II homologues that are also present in *P. falciparum* (PfHDAC2 and 3)^25–27^.

Based on this assumption, a series of peptoid-based HDACi were developed^5,6^. These compounds are classical HDAC inhibitors that have a cap-linker-zinc binding group structure with a peptoid-based cap group (*N*-alkyl glycine derivatives). Preclinical screens of these candidates identified potent activity against blood stages of *P. falciparum* laboratory strains 3D7 and Dd2 and against *P. berghei* liver stages with promising parasite selectivity indices^5,6^.

*In vitro* activity assessment of candidates against clinical *P. falciparum* isolates in early drug development can inform about the drug´s potency against parasite strains circulating in the target population in malaria endemic areas. *Plasmodium* parasites sampled from malaria patients are genetically very different from laboratory strains of *P. falciparum* that have been in *in vitro* culture for decades^28^. Additionally, the natural *P. falciparum* population is constantly exposed to host factors including antimalarial drug pressure and is therefore genetically highly diverse, and parasites may be intrinsically heterogenous in their susceptibility towards the molecule^29,30^. An additional layer of complexity results from clinical trials reporting different drug efficacies (of non-HDACi) against *P. falciparum* infections in adults and children^31–33^. These differences are mostly attributed to the partial immunity that is developed by the populations living in malaria endemic regions after multiple *P. falciparum* infections^34,35^. However, it has not been investigated if the parasites themselves isolated from children or adults show different drug susceptibility profiles in *in vitro* assays. Age-dependent immune responses that cause a difference in the number of *P. falciparum* strains co-infecting a single individual, also known as multiplicity of infection, could be one factor that provokes different susceptibility profiles *in vitro*.

Amongst the panel of previously published peptoid HDAC inhibitors, we selected 12 candidates with an IC50 below 100 nM against the laboratory strain 3D7 and different toxicity profiles for *in vitro* potency testing against *P. falciparum* isolates collected from infected individuals in Gabon, a country highly endemic for malaria^5,6,36–38^. We furthermore investigated the susceptibility of *P. falciparum* parasites isolated from children and adults towards standard antimalarial compounds and compared their activity profile.

## Results

In total, 85 clinical isolates were collected from 52 children and 33 adults with uncomplicated *P. falciparum* malaria in Gabon. Clinical isolates were tested for their susceptibility to 12 HDACi candidates, 1 approved HDACi cancer drug as comparator and 8 known antimalarial compounds. Of the 85 assays, 53 (33 from children, 20 from adults) tests fulfilled strict quality criteria for successful growth and were included into further analysis of the inhibitor concentrations. The median age (IQR) of children and adults included was 3 years (2–4 years) and 21 years (19–50 years), respectively. The median parasitemia (IQR) in children and adults was 25,000 parasites/µl (9,120–62,192 p/µl) and 3,933 parasites/µl (1,802–14,193 p/µl), respectively.

### *In vitro* activity of peptoid-based HDAC inhibitors against laboratory and clinical *P. falciparum* isolates

We assessed *in vitro* activity of 12 peptoid-based HDACi candidates against *P. falciparum* isolates obtained from children and adults. The panel includes molecules from two generations of synthesis, no. 1 series (1a, 1d, 1g, 1h, 1i, 1u, and 1v) and no. 2 series (2c, 2g, 2h, 2i and 2m) (see Supplementary Fig. 1)^5,6^. Compounds were also tested against 3D7 laboratory strains to confirm activity of new compound production lots (Table 1). Compound 1 u was the most active HDACi candidate with a molecular activity of approx. 13 nM against *P. falciparum* strains isolated from both, children and adults (see Table 1). Compounds 1a, 1d, 1h, 1v, 2g, 2h, and 2i had good antiplasmodial activities with IC50 values in the double digit nanomolar range in children and adults. The IC50 values of drug candidates showed a wide range of activity (Supplementary Fig. 2). Some HDACi (1a, 1g, 1h, 1v, 2c, 2g, and 2h) were at least 2-fold more active against the parasite strains obtained from children compared to adults’ strains, but differences did not reach the level of statistical significance.

**Table 1:** Median IC50 and interquartile range (IQR) of HDACi candidates against *P. falciparum* clinical isolates, and laboratory strains 3D7 and Dd2.

Compound ID	Median IC50 (IQR) in nM						SI
	Clinical isolates – children & adults combined^a^	Clinical isolates – children only^a^	Clinical isolates – adults only^a^	3D7^b^	3D7^c^	Dd2^c^	
1a	47.1 (15.9–386.9)	42.6 (20.2–175)	116 (11.7–910)	21.3	20 ± 10	12 ± 8	>1295
1d	21.9 (8.8–97.8)	21.6 (9.1–71.4)	25.9 (7.1–127)	8.7	11 ± 4	14 ± 6	>850
1g	167 (34.6–1022)	117 (34.1–1268)	326 (37.7–1020)	49.4	25 ± 18	31 ± 12	>1613
1h	38.4 (11.0–96.3)	26.3 (12.6–92.8)	73.7 (10.0–97.3)	23.3	9 ± 3	15 ± 6	>520
1i	174 (61.7–406)	126 (71.0–413)	215 (45.9–405)	54.9	21 ± 16	33 ± 15	>642
1u	13.6 (4.4–89.8)	14.7 (4.6–76.9)	13.1 (3.1–146)	12.1	4 ± 1	1 ± 1	>2496
1v	65.1 (28.7–220)	54.3 (26.6–95.9)	110 (38.9–316)	9.8	14 ± 4	14 ± 4	>499
2c	410 (152–966)	287 (168–861)	619 (130–1116)	150.4	95 ± 15	ND	>526
2g	47.8 (9.6–215)	31.2 (9.6–231)	113 (6.8–200)	40.7	8.8 ± 3.2	ND	1483
2h	26.9 (5.2–94.5)	17.8 (5.6–93.3)	35.6 (3.8–95.5)	11.8	5.2 ± 3.6	ND	889
2i	32.1 (17.7–59.1)	28.0 (17.5–44.0)	40.8 (21.9–71.0)	11.3	9.7 ± 2.9	ND	64
2m	348 (191–760)	351 (193–834)	337 (178–535)	209.5	87 ± 44	ND	234
SAHA	267 (172–392)	276 (166–395)	267 (187–372)	187.3	139 ± 73	146 ± 22	>15

^a^Median (IQR).

^b^Single measurement in duplicate at study start.

^c^Previously published data (mean IC50)^5,6^.

^d^SI Selectivity index: IC50 HepG2/IC50 3D7.

ND: not determined.

### Comparing drug susceptibility of *P. falciparum* strains obtained from children and adults

To investigate if parasites obtained from semi-immune adults are less fit and more susceptible to drug testing *in vitro*, assays were done with standard and well-characterized antimalarial compounds tested against *P. falciparum* isolates from children and adults. All compounds confirmed potency against Gabonese parasite strains including chloroquine (Table 2). No significant differences in median IC50s between isolates from children and adults were observed for any of the drugs.

**Table 2:** Median IC50 and interquartile range (IQR) of standard antimalarial compounds against *P. falciparum* clinical isolates obtained from children and adults.

Compound ID	Median IC50 (IQR) in nM			
	Clinical isolates – children & adults combined^a^	Clinical isolates – children only^a^	Clinical isolates – adults only^a^	P-value*
Chloroquine	15.4 (6.1–41.2)	12.3 (5.3–41.2)	15.7 (6.5–34.2)	0.853
Lumefantrine	2.8 (1.5–7.1)	3.3 (1.6–6.6)	2.4 (1.4–11.5)	0.662
Amodiaquine	2.2 (1.4–3.3)	2.8 (1.8–3.8)	1.7 (1.1–2.7)	0.009
Piperaquine	4.1 (2.8–7.2)	4.1 (2.8–7.5)	3.6 (2.8–5.8)	0.374
Pyronaridine	1.1 (0.4–2.1)	1.2 (0.4–2.3)	0.6 (0.4–1.7)	0.365
Ferroquine	1.8 (1.0–3.0)	1.9 (1.0–3.4)	1.4 (0.9–2.5)	0.159
Mefloquine	3.4 (2.2–5.1)	3.7 (2.2–5.5)	3.0 (2.3–5.1)	0.873
Atovaquone	0.3 (0.2–0.5)	0.4 (0.2–0.5)	0.3 (0.2–0.5)	0.686

^a^Median (IQR).

^*^Differences in activities of clinical isolates of adults and children were compared by Mann-Whitney U test.

### Quality control

Stability of dissolved test compounds during the study period of approximately 8 months was controlled by comparing IC50s against 3D7 assessed at project start and end. A fold increase of 1 was used to determine the stability of a compound at study end. Drug instability was not observed in any of the standard antimalarials or SAHA. Except for 1h, 2g and 2m, all HDACi candidates were at least 1.6-fold less active at the study end when controlled with 3D7 (Supplementary Fig. 3). Strains from children and adults were equally sampled over time (data not shown).

## Discussion

The development of new drugs and particularly of those with novel targets and modes of action is urgently needed to compete with the development of resistance by *P. falciparum* to current antimalarials. So far, mainly broad-spectrum HDACi have been investigated as potential antiplasmodial drugs, since several human HDAC homologues have been characterized in *P. falciparum*. Anti-cancer HDACi, already FDA approved, have been tested against *P. falciparum* and have been shown to effectively kill the parasites *in vitro* and also showed high activity against *P. berghei* in *in vivo* mouse models at sub-micromolar concentrations, but are known to cause a certain level of toxicity to human cells^6,18,20^. HDACi affect multiple eukaryotic cell functions, including non-histone related pathways where class IIb HDACs (e.g. hHDAC6) are involved^24^. Homologues of hHDAC6 proteins have been identified in *P. falciparum* and targeting class II HDACs might be a possibility to circumvent toxicity in humans^24^.

We tested the 12 most promising peptoid-based HDACi candidates against clinical *P. falciparum* isolates from Gabon and confirmed previous results in laboratory strains which showed the HDAC6 inhibitor 1u, *N*-(2-(Cyclohexylamino)-2-oxoethyl)-*N*-(4-(hydroxycarbamoyl)benzyl)-4-isopropylbenzamide, to be the most potent HDACi with a mean IC50 of 4 nM and high selectivity towards the parasites^5^. Here, our results also showed compound 1u to be the most active HDACi against clinical *P. falciparum* isolates. The high drug activity of 1u may have been obtained by replacement of the *N*, *N*-dimethylamino group, from molecule 1h, with a less polar isopropyl group^5^. The results of our investigation in clinical isolates resemble those previously obtained in laboratory strains^6,12^, confirming that these compounds are equally active against a parasite population of high genetic diversity under selection pressure by the currently used antimalarial drugs^30,37^. All compounds were more active than the comparator broad-spectrum HDACi SAHA (vorinostat), which we found to have an IC50 similar to that reported in the literature^15,39^. HDACi candidates, including potent compound 1u, seem to suffer from chemical instability in solution that is less pronounced for SAHA. Loss in activity over time may also explain the observed wide range of IC50 data, that otherwise could be interpreted as suggesting towards intrinsic parasite resistance.

Differences in *in vivo* drug activities against *P. falciparum* infections between children and adults are usually attributed to the well-known acquisition of immunity after repeated infections in high endemic regions^32–34^. There is evidence that multiplicity and diversity of strains differs between adults and children^40–42^. However, to the best of our knowledge, there has been no study that specifically looked at differences in antiplasmodial *in vitro* drug activity between children and adults. Overall, our results suggest no significant differences in *in vitro* activity of HDACi and standard antimalarials in *P. falciparum* strains collected from children and adults. We found only amodiaquine to potentially show such a difference, a drug to which the parasite population is constantly exposed, although this finding did not hold under correction for multiple testing. Whether this difference is a true finding has to be further examined in future investigations. Overall, however, our results suggest no significant differences in the *in vitro* activity of either HDACi or standard antimalarials against *P. falciparum* strains collected from adults and children.

The standard antimalarials we tested were all highly active against clinical *P. falciparum* isolates from Gabon with no observed resistance except in chloroquine, to which 13 outliers showed an IC50 greater than 40 nM. First line treatment in Gabon changed from chloroquine to the artemisinin combination therapies (ACTs) artesunate-amodiaquine or artemether-lumefantrine in 2003^43^. Despite this change, chloroquine resistance appears to remain high, but a tendency towards declining chloroquine resistance can be extrapolated from the *in vitro* data^43–46^. This might indicate that the full reversal of chloroquine sensitivity can be observed in the future, as has occurred in other malaria endemic regions^47^. Only a few outliers were present for the other standard antimalarials, which may not necessarily be attributed to resistance. Amodiaquine and lumefantrine are first-line partner drugs to artemisinin-derivatives in Gabon, but fortunately signs of resistance cannot be detected^48^.

Our analysis of clinical *P. falciparum* isolates from Gabon confirm the results from previous work showing HDACi candidate 1u to be highly active and indeed more potent than the comparator cancer drug SAHA. These inhibitors targeting class II HDAC proteins are potential lead structures for further development as antimalarial chemotherapeutics with promising selectivity towards *Plasmodium* parasites, but require improvement of chemical stability. Differences in the immune status to malaria between adults and children seemed not to affect the observed drug potency against *P. falciparum*.

## Methods

### Clinical isolate sampling

In total, 85 participants with uncomplicated malaria were enrolled between October 2017 and June 2018 residing in Lambaréné and surrounding villages in Gabon. Informed consent was obtained from study participants or the legal representative, if minor. The study was approved by the Institutional Ethics Committee of the Centre de Recherches Médicales de Lambaréné (CERMEL) with the number CEI-CERMEL015/2015. Inclusion criteria were written informed consent, age either 1 to 5 years (children cohort) or 18 years and older (adult cohort), and *P. falciparum* monoinfection with a parasitemia above 1000 parasites/µl assessed by Giemsa-stained thick blood smear. To obtain clinical *P. falciparum* isolates, a venous blood sample was taken in a lithium heparin tube/EDTA tube and processed in the *in vitro* drug sensitivity assay within approximately 6 hours. All methods were performed in accordance with relevant guidelines and regulations.

### Parasite culture

*Plasmodium falciparum* laboratory strain 3D7 (chloroquine-sensitive) was maintained in continuous *in vitro* culture as previously described^49^. Parasites were kept in complete culture medium (RPMI 1640, 25 mM 4-(2-hydroxyethyl) piperazine-N′-(4-butanesulfonic acid), 2 mM L-glutamine, 50 μg/mL gentamicin, and 0.5% w/v albumax) at 37 °C, at 2.5% hematocrit in a candle jar with daily change of medium. Synchronization was performed by 5% sorbitol twice a week^50^.

### Compounds

All compounds were dissolved in sterile DMSO if not otherwise stated. In total,12 candidate HDAC inhibitors were tested and 1g, 1h, 1i, 1u, 2c, 2g, 2i, and 2m were dissolved to reach a stock concentration of 25 mM and 1a, 1d, 1v, and 2h were prepared at 100 mM (chemical structures see Supplementary Fig. 1). SAHA (Hycultect), an approved HDAC inhibitor served as a control and was dissolved at 100 mM in DMSO. All comparator antimalarial compounds were obtained from Sigma-Aldrich if not otherwise stated. Lumefantrine, mefloquine hydrochloride, ferroquine (Sanofi-Synthelabo), were prepared at 12.5 mM stock concentration; 100 mM stocks were made for amodiaquine dihydrochloride dihydrate, pyronaridine tetraphosphate, piperaquine tetraphosphate tetrahydrate was dissolved at 6.25 mM; and atovaquone (GlaxoSmithKline) at 25 mM. Chloroquine diphosphate salt was dissolved in double-distilled water at 100 mM. All stocks were freshly prepared for the study and stored at −20 °C. Maximum concentration of solvent DMSO in the *in vitro* assay was 0.01% and did not interfere with parasite growth in pilot experiments.

### Drug sensitivity assay

Drug sensitivity assays were performed according to standard procedures^46^. Briefly, 96 well-plates were pre-dosed with a threefold serial dilution of the respective drug in complete culture medium to obtain the following range of concentrations: chloroquine, 1.2 to 1000 nM; lumefantrine, 0.4 to 1000 nM; amodiaquine, 0.4 to 100 nM; piperaquine, 0.3 to 250 nM; pyronaridine, 0.02 to 50 nM; ferroquine, 0.08 to 200 nM; mefloquine, 0.7 to 500 nM; atovaquone, 0.02 to 20 nM; 1a, 4.1 to 10000 nM, 1d, 1.2 to 3000 nM; 1g, 12.3 to 10000 nM; 1h, 0.34 to 750 nM; 1i, 12.3 to 10000 nM; 1u, 0.34 to 750 nM; 1v, 2.7 to 2000 nM; 2c, 12.3 to 15000 nM; 2g, 0.34 to 750 nM; 2h, 1.2 to 1000 nM; 2i, 0.41 to 300 nM; 2m, 6.9 to 5000 nM; SAHA, 12.3 to 15000 nM. Ring-stage parasites from the laboratory strain 3D7 and clinical *P. falciparum* isolates were adjusted to a parasitemia of 0.05% with 0^+^ erythrocytes and the hematocrit was set to 1.5% in a total volume of 225 µl per well. After 72 hours of incubation at 37 °C, plates were freeze-thawed three times and analyzed by measurement of *P. falciparum* histidine-rich protein 2 (HRP2) with an enzyme-linked immunosorbent assay (ELISA)^51^. Only assays with successfully grown and propagating parasites reflected by a 1.5 OD increase between full and no inhibition within the 72 hours were included in the IC50 analysis. All experiments were done in duplicates. To control quality and stability, all compounds were additionally tested against the 3D7 laboratory strain once before and three times after testing of clinical isolates.

### Statistics

Individual inhibitory concentrations were determined by non-linear regression analysis of log-concentration-response curves using the drc-package v3.0-1 of R version 3.4.2. Data for the clinical isolates was presented using the median 50% inhibitory concentration (IC50) and the interquartile range (IQR). Correlations between IC50 values of clinical isolates of children and adults of the different standard antimalarials and HDACi were calculated using the Mann-Whitney U (nonparametric) test in JMP v14.0.0 software. IC50 values, calculated by R, to be over the highest tested drug concentration were substituted with the highest tested concentration value if parasites were dead at the highest tested drug concentration (indicated by a low OD value of confirmed dead parasites from the same patient).

### Ethics approval and consent to participate

The study was approved by the Institutional Ethics Committee (CEI) of CERMEL with the number CEI-CERMEL015/2015.

## Supplementary information

Supplementary Figures

## Data Availability

The datasets generated during and/or analyzed during the current study are available from the corresponding author on reasonable request.
