# Supplementary material for: Histone deacetylase inhibitors with high in vitro activities against Plasmodium falciparum isolates collected from Gabonese children and adults
Source: Sci Rep. 2019 Nov 22;9:17336. doi: 10.1038/s41598-019-53912-w (PMC6874535; doi:10.1038/s41598-019-53912-w)
Supplement: Supplementary file 1 — Supplementary Figures [file 41598_2019_53912_MOESM1_ESM.pdf]

Title: Histone deacetylase inhibitors with high in vitro activities against *Plasmodium falciparum* isolates collected from Gabonese children and adults

Authors: Erik Koehne [a,b*], Andrea Kreidenweiss [a,b*], Rella Zoleko Manego[b,c], Matthew McCall[b], Ghyslain Mombo-Ngoma[b,c], Marcel Karl Walter Mackwitz[d] , Finn K. Hansen[d], Jana Held[a,b]

Author Affiliations:

[a] Institute of Tropical Medicine, University Hospital Tübingen, Wilhelmstrasse 27, D-72074 Tübingen, Germany;

[b] Centre de Recherches Médicales de Lambaréné, B.P. 242, Lambaréné, Gabon;

[c] Department of Tropical Medicine, Bernhard Nocht Institute for Tropical Medicine & I. Dep of Medicine, University Medical Centre- Hamburg-Eppendorf, Bernhard-Nocht-Strasse 74, D-20359 Hamburg, Germany;

[d] Institute of Pharmacy, Medical Faculty, University of Leipzig University, Brüderstrasse 34, D-04103 Leipzig, Germany

*These authors contributed equally to this work

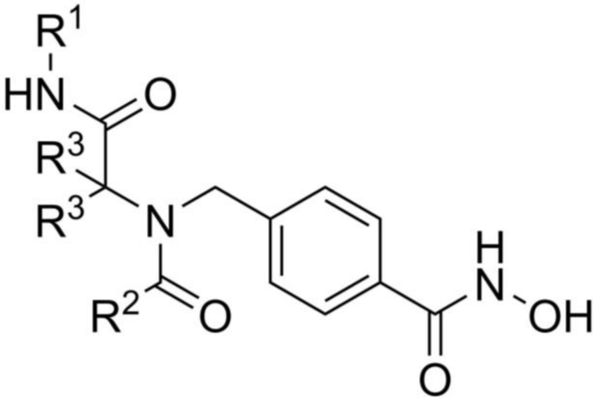

**Compound ID**	**R^1**	**R^2**	**R^3**
**1a**	*t*-Bu	3,4-Me-Ph	H
**1d**	*t*-Bu	4-Me_2N-Ph	H
**1g**	*c*-Hex	1-Naphthyl	H
**1h**	*c*-Hex	4-Me_2N-Ph	H
**1i**	*c*-Hex	3,5-Me-Ph	H
**1u**	*c*-Hex	4-*i*-Pr-Ph	H
**1v**	*n*-Bu	4-Me_2N-Ph	H
**2c**	*c*-Hex	4-Me_2N-Ph	Me
**2g**	Bn	4-*t*-Bu-Ph	H
**2h**	Bn	4-*i*-Pr-Ph	H
**2i**	Bn	4-Me_2N-Ph	H
**2m**	4-F-Bn	3,5-Me-Ph	H

Supplementary Figure 1: Chemical structures of tested compounds

Chemical structures have already been published [5,6]

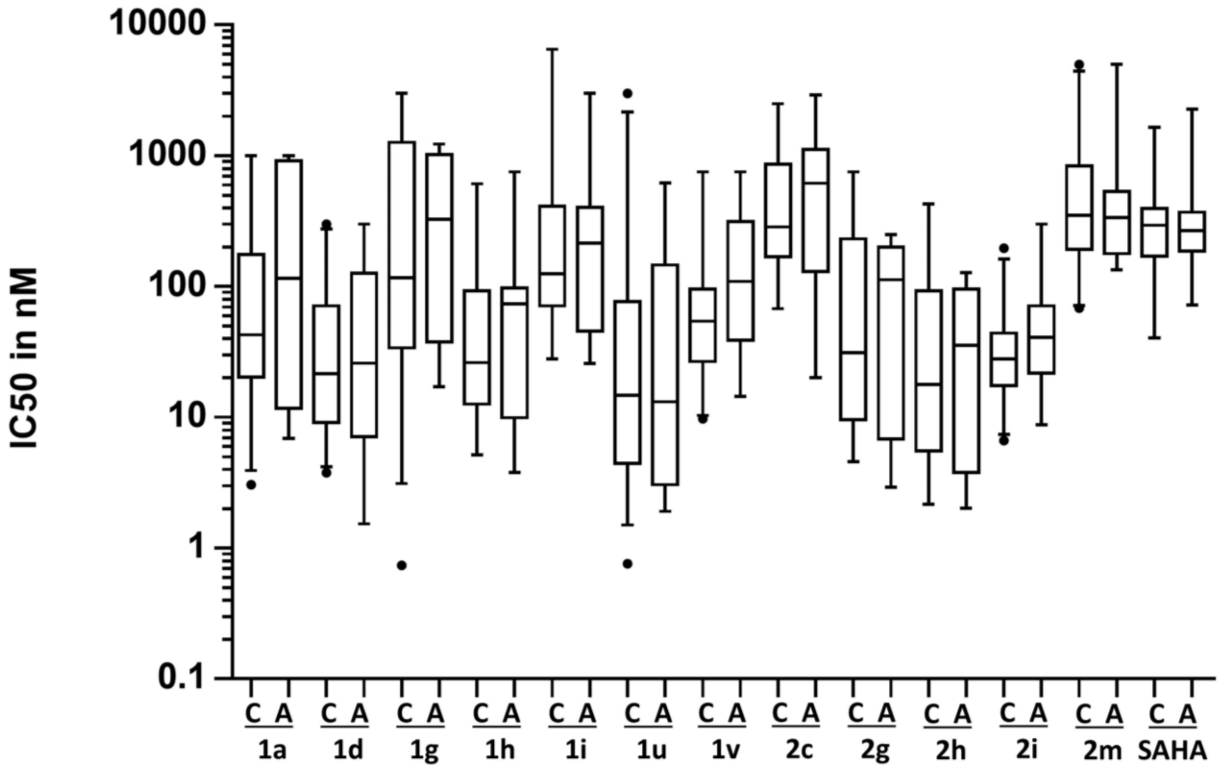

Supplementary Figure 2: Distribution of IC50 values

Distribution of IC50 values of HDACi candidates against *P. falciparum* isolates obtained from children (C) and adults (A). SAHA: suberoylanilide hydroxamic acid (Vorinostat). Box plots show median IC50 and 25% and 75% quartiles, respectively. Whiskers extend to the 5th and 95th percentiles. Dots outside the whiskers are outlier IC50 values.

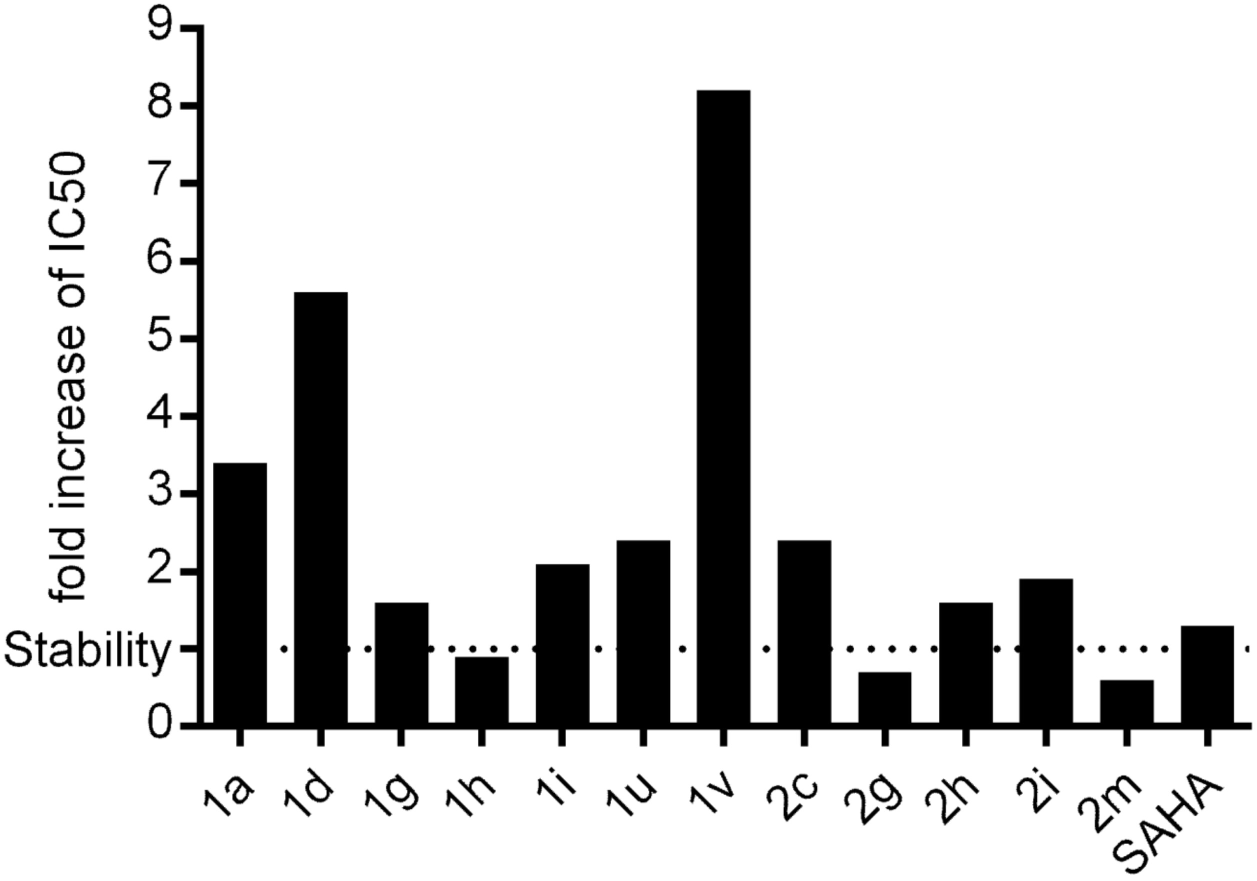

Supplementary Figure 3: Stability of HDACi candidates

Fold increase of IC50 at study end determined in *P. falciparum* 3D7. Fold increase (IC50 at study end/IC50 at study start) of 1 represents stability of compound at study end.
